# Effect of High Dietary Tryptophan on Intestinal Morphology and Tight Junction Protein of Weaned Pig

**DOI:** 10.1155/2016/2912418

**Published:** 2016-06-06

**Authors:** Myrlene Carine B. Tossou, Hongnan Liu, Miaomiao Bai, Shuai Chen, Yinghua Cai, Veeramuthu Duraipandiyan, Hongbin Liu, Tolulope O. Adebowale, Naif Abdullah Al-Dhabi, Lina Long, Hussain Tarique, Abimbola O. Oso, Gang Liu, Yulong Yin

**Affiliations:** ^1^Key Laboratory of Agro-Ecological Processes in Subtropical Region, Institute of Subtropical Agriculture, Chinese Academy of Sciences, Hunan Provincial Engineering Research Center of Healthy Livestock, Scientific Observing and Experimental Station of Animal Nutrition and Feed Science in South-Central, Ministry of Agriculture, Hunan Co-Innovation Center of Animal Production Safety, Hunan 410125, China; ^2^University of Chinese Academy of Sciences, Beijing 100049, China; ^3^China Animal Disease Control Center, Beijing 102600, China; ^4^Department of Botany and Microbiology, Addiriyah Chair for Environmental Studies, College of Science, King Saud University, P.O. Box 2455, Riyadh 11451, Saudi Arabia; ^5^Department of Animal Nutrition, College of Animal Science and Livestock Production, Federal University of Agriculture, PMB 2240, Abeokuta, Nigeria

## Abstract

Tryptophan (Trp) plays an essential role in pig behavior and growth performances. However, little is known about Trp's effects on tight junction barrier and intestinal health in weaned pigs. In the present study, twenty-four (24) weaned pigs were randomly assigned to one of the three treatments with 8 piglets/treatments. The piglets were fed different amounts of L-tryptophan (L-Trp) as follows: 0.0%, 0.15, and 0.75%, respectively, named zero Trp (ZTS), low Trp (LTS), and high Trp (HTS), respectively. No significant differences were observed in average daily gain (ADG), average daily feed intake (ADFI), and gain: feed (G/F) ratio between the groups. After 21 days of the feeding trial, results showed that dietary Trp significantly increased (*P* < 0.05) crypt depth and significantly decreased (*P* < 0.05) villus height to crypt depth ratio (VH/CD) in the jejunum of pig fed HTS. In addition, pig fed HTS had higher (*P* < 0.05) serum diamine oxidase (DAO) and D-lactate. Furthermore, pig fed HTS significantly decreased mRNA expression of tight junction proteins occludin and ZO-1 but not claudin-1 in the jejunum. The number of intraepithelial lymphocytes and goblet cells were not significantly different (*P* > 0.05) between the groups. Collectively, these data suggest that dietary Trp supplementation at a certain level (0.75%) may negatively affect the small intestinal structure in weaned pig.

## 1. Introduction

The postweaning period represents a delicate transitional phase in pig's life. The numerous stresses to their endocrinology, metabolism, and physiology that piglets experience following weaning are reflected in homeostatic changes to their bodies. The gastrointestinal tract is particularly responsive to stressors. Weaning is known to compromise the digestive, absorptive, and secretory capacity of the small intestine which can cause morphological and histological changes of the small intestine [1–4]. In addition, weaning induces a deleterious effect on intestinal barrier function [5, 6].

Tryptophan has a potential role to facilitate stress adaptation of animals and human through increasing hypothalamic serotonin (5-hydroxytryptamine, 5-HT) level [7, 8]. Several studies have shown that dietary Trp may reduce stress hormone [7, 9, 10], reduce aggressive behaviors [11], and improve growth performance [8] in weaned pigs. As the precursor of serotonin, tryptophan is also known to play an essential role in the regulating several physiological function, such as motility (in duodenal and ileal), secretion and sensitivity [12], and intestinal permeability [13, 14] in the gastrointestinal tract. However, little is known about the effect of dietary Trp on intestinal epithelial cells growth and health, as well as intestinal epithelial tight junction proteins in weaned piglets [7, 15] (Table 5). This study was designed to evaluate the effects of dietary Trp on intestinal epithelial morphology and mRNA level of tight junction proteins in weaned pig.

## 2. Materiel and Methods

### 2.1. Experimental Animals and Diets

The study was approved by the Animal Care and Use Committee of the Chinese Academy of Sciences and performed according to the Chinese Guidelines for Animal Welfare. This animal experiment was conducted at technology innovation platform for national research institutions located in Hunan province “Experimental Station for Healthy Production of Livestock and Poultry & Environmental Control.” Thirty-six healthy piglets of similar body weight (8.26 ± 0.15 kg) (landrace × large white) from different litters were obtained from a local farm and transported to the experimental site of the Institute of Subtropical Agriculture. Piglets were weighed and allocated to individual pens in the same room. The piglets were fed mash feed. Diets were formulated to approximately meet or exceed the nutrient requirements of growing pigs as suggested by NRC 2012 [16]. The isonitrogenous diets were formulated and supplemented with different amounts of L-tryptophan (L-Trp) as follows: 0.0%, 0.15, and 0.75%, respectively, named zero Trp (ZTS), low Trp (LTS), and high Trp (HTS). All of the diets were based on corn and soybean meal. Proteins from whey powder and fishmeal were kept constant. The diets were balanced for the same level of crude protein. The compositions of the diets are shown in Table 1.

The piglets were housed individually in an environmentally controlled nursery with hard-plastic slatted flooring. The piglets were exposed to a 3-day adaptation period before being allocated to one of three dietary treatments. For 21 days, the piglets were given ad lib access to water and their respective diet. Each day, surplus feed and waste were collected and weighed. This was then dried at 100°C for several hours and weighed again to determine the initial dry matter content and calculate the feed intake. Each piglet's body weight was monitored weekly. The feed conversion rate was calculated from the body weight and feed intake data.

### 2.2. Sample Collection

At day 22, after pigs were feed-deprived overnight, approximately 10 mL of blood was collected from jugular vein and serum samples were obtained by centrifugation at 2,000 ×g for 10 min at 4°C. These samples were immediately stored at −80°C for amino acids, D-lactase, and diamine oxidase determination. After blood sample collection, pigs were euthanized by electrical stunning and exsanguination. The gastrointestinal tract was removed and immediately dissected, with the small intestine being divided at the duodenum, jejunum, and ileum. Using sterile instruments, approximately 20 cm of intestinal tissue was removed from the center of each of these sections. Tissue samples were flushed with ice-cold saline to recover mucosa, yielding approximately 5 g from each tissue. The mucosa samples were straightaway frozen in liquid nitrogen and stored at –80°C until RNA was ready to be collected. To determine the morphology of the small intestine mucosa and to evaluate the proliferation of crypt cells, a 2 cm segment from each small intestine section was cut and fixed in 4% formaldehyde.

### 2.3. Intestinal Morphology and Crypt Cell Proliferation

Samples of the duodenum, jejunum, and ileum were fixed in formalin and then embedded in paraffin. Using a microtome, cross sections of the samples were cut to an approximate thickness of 5 *μ*m and stained with haematoxylin and eosin. For each section, the density of goblet cells and lymphocytes together with the height of the villus (VH) and depth of crypts (CD) was calculated using computer-assisted microscopy (Nikon, ECLIPSE E200, Tokyo, Japan). To assess VH and CD consistently, measurements were made of the distance between the tip of the villus to the mouth of the crypt (VH) and the crypt mouth to the crypt base (CD). The ratio of VH to CD (VH/CD) was determined.

### 2.4. Serum Large Neutral Amino Acids Analysis

The serum obtained from the blood samples was used for large neutral amino acids determination. Isotope dilution liquid chromatography-tandem mass spectrometry was used to analyze serum amino acids. Analysis was conducted by Beijing Amino Medical Research Co., Ltd., Beijing, China.

### 2.5. Serum D-Lactate and Diamine Oxidase Levels in the Plasma

Serum D-lactate content was measured by a commercial kit (Sino-German Beijing Leadman Biotech Ltd., Beijing, China) and Beckman CX4 Chemistry Analyzer (Beckman Coulter, Brea, CA). The activities of the serum diamine oxidase were determined using kits according to the user's manual (Nanjing Jiancheng Bioengineering Institute, Nanjing, China).

### 2.6. Relative Quantification of mRNA Expression

The abundance of occludin, zonula occludens-1 (ZO-1), and mRNA in the ileal mucosa was determined by RT-PCR.

TRIzol reagent (Invitrogen, Carlsbad, CA, US) was used in accordance with the manufacturer's instructions to isolate RNA from the sample of liquid nitrogen-pulverised ileal mucosa. To ensure the integrity of the RNA, it was checked using 1% agarose gel electrophoresis and 10 *μ*g/mL ethidium bromide stain. The quantity and quality of the RNA were analyzed by UV/Vis spectroscopy using a spectrophotometer (NanoDrop ND-1000; Thermo Fisher Scientific, Germany).

Complementary DNA (cDNA) was synthesised using 5x PrimeScript Buffer 2 and PrimeScript reverse transcriptase Enzyme Mix 1 (Takara Biotechnology (Dalian) Co., Ltd., Dalian, China). To assess gene expression, the ensuing cDNA was diluted and used as a PCR template. The reaction was carried out in 10 *μ*L of PCR solution (ABI Prism 7700 Sequence Detection System); quantitative PCR (qPCR) analyses were conducted (ABI 7900HT Fast Real-Time PCR System; Applied Biosystems, Carlsbad, CA, USA) using 5 *μ*L SYBR Green mix, 1 *μ*L cDNA (diluted ×4), 0.2 *μ*L each of forward and reverse primers (details available in Table 2), 3.6 *μ*L H_2_O, and 0.2 *μ*L ROX Reference Dye (50x). Samples were exposed to predenaturation of 10 s at 95°C followed by 40 cycles of amplification; each cycle consisted of 5 s at 95°C and 20 s at 60°C. This was followed by a melting curve stage of a heating rate of 0.1°C/s^−1^ and melt temperature of 60°C to 90°C. GADPH was used as the reference gene. The relative expression of mRNA was calculated for the treatment and control groups as a ratio of target genes to GAPDH using *R* = 2^−ΔΔCt^, where ΔΔCt is the difference between Ct^(gene  of  interest)^ − Ct^(GAPDH)^ of the treatment condition and Ct^(gene  of  interest)^ − Ct^(GAPDH)^ of the control condition.

### 2.7. Statistical Analysis

All statistical analysis was performed using the Analysis of Variance (ANOVA) with the comparison of means by Duncan's Multiple Comparison Test using IBM SPSS statistics version 22. A *P* value less than 0.05 was considered to be significant.

## 3. Results

### 3.1. Growth Performance

The growth performance is presented in Table 3. No significant differences (*P* > 0.05) were observed among treatments in daily gain, feed intake, and gain/feed. However, ADG was numerically improved by 8% and 11% during d7–d14 and 16% and 36% during d14–d21, respectively, by low Trp supplementation (LTS) and high Trp supplementation Trp (HTS) compared to zero Trp supplementation (ZTS).

### 3.2. Plasma Large Neutral Amino Acids

Table 4 showed that dietary Trp supplementation significantly increased (*P* < 0.05) Trp and Trp/LNAA (large neutral amino acids) concentration in the plasma from the pig fed LTS and HTS compared with those fed ZTS. The concentration of valine (Val) and isoleucine (Ile) significantly decreased (*P* < 0.05) in plasma from pig fed dietary Trp but Leu (leucine), Tyr (tyrosine), and Phe (phenylalanine) were the same between control and treatment groups.

### 3.3. Effect of Tryptophan on Pig Intestinal Morphology

The morphology of the duodenum, jejunum, and ileum at 21 days of the experiment is shown in Table 4.

The results showed that pig fed HTS significantly increased (*P* < 0.05) CD and decreased VH/CD (*P* < 0.05) in the jejunum compared to ZTS and LTS. No significant differences (*P* > 0.05) were observed for villi height between groups.

### 3.4. Effect of Trp on Intestinal Barrier Function

The effect of dietary Trp on serum DAO activity and D-lactate content is shown in Table 6. There was no significant increase in serum DAO and D-lactate levels between the control and LTS group. Pig fed HTS had significantly increased DAO and D-lactate level compared to ZTS and LTS.

**Figure 1:**
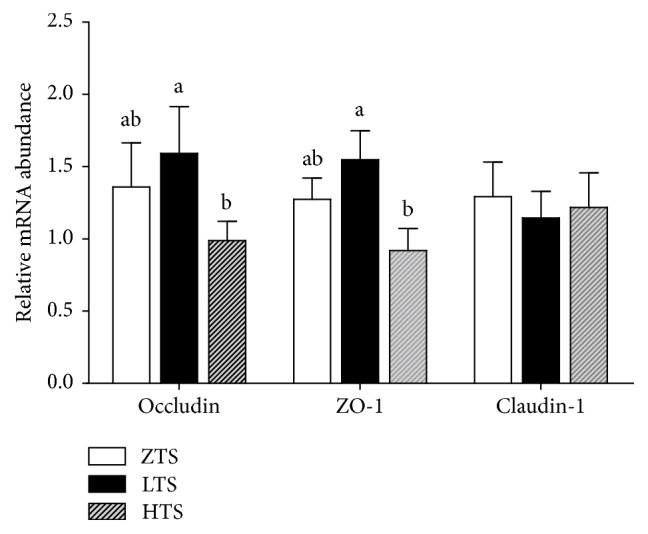
mRNA abundance in the jejunum. ^a, b^Values with different letters within the same row are different (*P* < 0.05).

As shown in Figure 1, HTS significantly decreased mRNA level of occludin and ZO-1 in the jejunum of pig fed HTS compared to LTS and ZTS. mRNA expression of claudin-1 was the same between the groups.

## 4. Discussion

In this study, we found that plasma Trp was significantly increased in the plasma of pigs fed dietary Trp. This could be an indicator for Trp uptake in the small intestine. Similar results were found by Koopmans et al.; they reported that dietary Trp supplementation increased plasma Trp concentration by two times [17]. The result of this study is in accordance with Martínez-Trejo et al. and Li et al. who reported that Trp supplementation affects behavior but did not have any effect on productive performance (FI, DG, and FCR) [18, 19]. The small intestinal histomorphology including villus height, crypt depth, and their ratio is one of important indications of gut health in pigs. A healthy gut has a high villus height to crypt depth ratio. In the present study, pig fed HTS had deeper crypt and lower villus height to crypt depth ratio in the jejunum. These results parallel the findings of Koopmans et al., who suggested that dietary (0.5 g/kg Trp) in nursery pig tends to increase villus height at certain sites along the small intestine [17]. Reduced villus height and increased crypt depth (reduced ratio) lead to increased endogenous secretion and reduced nutrient absorption, disease resistance, and performance [18]. Deep crypts are associated with a high cell turnover. Villi are continually renewed as vulnerable to ordinary sloughing as well as pathogenic assault and pathogen-initiated inflammation [19]. Equally, the high level of activity associated with deeper crypts is costly in energy and nutrients; to obtain adequate nutrients needed for higher mucosal growth may divert energy that is needed for performance. This could be one of the reasons why pig fed HTS had no significant improvement on growth performance in this study.

D-Lactic acid is the final end-product of bacterial fermentation in the gut. Increased plasma D-lactic acid levels reflect changes in intestinal permeability [20]. DAO is an enzyme that serves as an indicator of intestinal epithelial integrity. The intestinal mucosa damaged leads to an increase in serum DAO level [21]. In the present study, HTS increased D-lactate content and DAO activity in the serum of pig fed HTS at d21 after weaning which may indicate impaired intestinal integrity. Tight junction proteins are the principal determinants of epithelial and endothelial paracellular barrier functions [22, 23]. To maintain the integrity of the intestinal barrier, epithelial cells are joined by tight junction proteins, such as claudin, occludin, and ZO-1 [24]. By allowing ions, small molecules, nutrients, and water to cross the cell, whilst restricting pathogens, tight junction proteins mediate the epithelial barrier. The results of this study showed that HTS significantly decreased mRNA expression of tight junction proteins ZO-1 and occludin which was consistent with the increased concentration of serum D-lactate and DAO in the plasma. This suggests that high dietary supplementation of Trp may compromise the intestinal barrier integrity in weaned pig. According to Jiang et al., Trp deficiency or excess could cause antioxidant system disruption and change tight junction protein transcription abundances in the fish gill [25]. One of the most important factors in the growth of intestinal microbiota is gut motility [26]. Intestinal motility, together with mucus secretions, provides another epithelium defense mechanism by forcing pathogens and toxins along the gut lumen to be eventually voided. However, increase in intestinal motility and secretion in the small intestine may lead to intestinal permeability [27]. According to Kojima et al., 5-HT, released from damaged enterochromaffin cells resulting from an allergic reaction, increases the strength and rate of intestinal peristalsis and then contributes to diarrhoea [28]. Overall, our results showed that, at moderate supplementation (0.15%), dietary Trp did not affect pig performance. However, at a much higher level (0.75%), dietary Trp could negatively affect intestinal morphology and tight junction proteins in weaned pigs. The change in morphological and tight junction expression in pig fed high dietary Trp (0.75%) supplementation in the present study provides new information regarding the potentials for using Trp in weaned pigs.

## Figures and Tables

**Table 1:** Ingredient and chemical composition of experimental diets.

Item	Dietary Trp supplementation%		
	ZTS	LTS	HTS
*Ingredients%*			
Corn	64.61	64.96	65.37
Soybean meal	19.50	19.00	17.30
Whey powder	4.50	4.50	4.50
Fish meal	5.50	5.50	5.50
Soybean oil	2.40	2.40	3.00
Lysine	0.55	0.55	0.60
Methionine	0.18	0.18	0.20
Threonine	0.18	0.18	0.20
Tryptophan	0.00	0.15	0.75
DCP	0.76	0.76	0.76
Limestone powder	0.52	0.52	0.52
Salt	0.30	0.30	0.30
^2^Premix	1.00	1.00	1.00
Total	100.00	100.00	100.00
*Nutrient levels%*			
DE (MJ/kg)	14.65	14.63	14.62
CP	18.08	18.05	18.05
Lysine	1.23	1.23	1.23
Methionine + cysteine	0.68	0.68	0.68
Threonine	0.73	0.73	0.73
Tryptophan	0.15	0.30	0.90
Leucine	1.25	1.25	1.25

^1^ ZTS: zero Trp supplementation (0% Trp); LTS: low Trp supplementation (0.15% Trp); HTS: high Trp supplementation (0.75% Trp).

^2^The following minerals and vitamins per kilogram were provided in the premix (as-fed basis): Zn (ZnO), 50 mg; Cu (CuSO_4_), 20 mg; Mn (MnO), 55 mg; Fe (FeSO_4_), 100 mg; I (KI), 1 mg; Co (CoSO_4_), 2 mg; Se (Na_2_SeO_3_), 0.3 mg; vitamin A, 8,255 IU; vitamin D3, 2,000 IU; vitamin E, 40 IU; vitamin B_1_, 2 mg; vitamin B_2_, 4 mg; pantothenic acid, 15 mg; vitamin B_6_, 10 mg; vitamin B_12_, 0.05 mg; vitamin PP, 30 mg; folic acid, 2 mg; vitamin K_3_, 1.5 mg; biotin, 0.2 mg; choline chloride, 800 mg; and vitamin C, 100 mg.

**Table 2:** 

Gene	Accession number	Primer sequence 5′-3′
Occludin	NM_001163647.1	F: TCCTGGGTGTGATGGTGTTC
		R: CGTAGAGTCCAGTCACCGCA

Zonula occludens-1	XM_003353439.2	F: AAGCCCTAAGTTCAATCACAATCT
		R: ATCAAACTCAGGAGGCGGC

Claudin-1	NM_001244539.1	F: AGAAGATGCGGATGGCTGTC
		R: CCCAGAAGGCAGAGAGAAGC

**Table 3:** Effect of dietary tryptophan on pigs growth performance.

Item	Dietary L-tryptophan				
	ZTS	LTS	HTS	SEM	*P* value
*Initial BW, Kg*	8.26	8.25	8.26	0.15	0.62
*Final BW, Kg*	14.54	14.89	15.53	0.49	0.46
*ADG, g*					
d0– d7	321.43	298.21	285.71	15.36	0.40
d7– d14	244.64	264.29	273.81	22.00	0.63
d14–d21	330.36	385.71	450.00	28.46	0.12
d1–d21	298.81	316.07	336.51	18.61	0.46
*AFI, g*					
d0–d7	464.29	419.64	405.36	00.00	00.00
d7–d14	496.43	500.00	497.62	25.59	0.96
d14–d21	671.43	703.57	726.19	31.25	0.53
d1–d21	544.05	541.07	562.36	18.16	0.67
*Gain: feed*					
d0–d7	0.69	0.71	0.62	0.04	0.33
d7–d14	0.49	0.54	0.53	0.02	0.48
d14–d21	0.49	0.52	0.62	0.03	0.16
d1–d21	0.54	0.58	0.59	0.02	0.55

ZTS: zero Trp supplementation (0.00% Trp); LTS: low Trp supplementation (0.15% Trp); HTS: high Trp supplementation (0.75% Trp).

**Table 4:** Large neutral amino acids in the plasma.

Item	Dietary L-tryptophan (%)				
	ZTS	LTS	HTS	SEM	*P* value
Trp	2.16^b^	5.48^a^	7.22^a^	0.59	0.006
Val	17.06^a^	10.67^b^	11.13^b^	0.90	0.006
Ile	10.91^a^	8.29^b^	7.37^b^	0.54	0.02
Leu	18.12	16.16	16.14	0.60	0.33
Tyr	6.77	7.16	5.93	0.44	0.54
Phe	15.46	14.23	16.2	0.48	0.25
LNAA	68.33	56.50	56.76	2.52	0.08
Trp/LNAA	3.18^b^	9.70^a^	11.12^a^	0.01	0.002

^a,b^Values with different letters within the same row are different (*P *< 0.05).

SEM: standard error mean; ZTS = 0.00%; LTS = 0.15%; HTS = 0.75%.

**Table 5:** Effect of dietary tryptophan supplementation on intestinal morphology.

Item	Dietary L-tryptophan (%)				
	ZTS	LTS	HTS	SEM	*P* value
*Duodenum*					
Villus height (*μ*m)	384.79	386.67	428.00	9.90	0.15
Crypt depth (*μ*m)	126.09	134.39	152.00	7.98	0.44
VH/CD	3.09	3.08	2.95	0.14	0.90
Goblet cell (unit)	20.00	24.57	19.33	1.57	0.22
Lymphocyte count (unit)	259.88	243.29	241.17	9.15	0.46
*Jejunum*					
Villus height (*μ*m)	350.99	299.48	336.06	12.34	0.22
Crypt depth (*μ*m)	116.20^b^	103.29^b^	152.44^a^	7.80	0.02
VH/CD	3.11^a^	2.98^a^	2.27^b^	0.12	0.004
Goblet cell (unit)	10.75	14.75	11.13	1.04	0.142
Lymphocyte count (unit)	281.13	302.13	296.38	8.20	0.34
*Ileum*					
Villus height (*μ*m)	266.96	309.95	300.11	8.72	0.1
Crypt depth (*μ*m)	106.77	104.78	138.73	7.48	0.12
VH/CD	2.701^ab^	3.12^a^	2.25^b^	0.14	0.09
Goblet cell (unit)	23.71	20.13	17.29	2.03	0.24
Lymphocyte count (unit)	257.57	304.25	299	11.07	0.1

^a,b^Values with different letters within the same row are different (*P *< 0.05).

ZTS: zero Trp supplementation (0% Trp); LTS: low Trp supplementation (0.15% Trp); HTS: high Trp supplementation (0.75% Trp).

**Table 6:** Plasma DAO and diamine oxidase levels.

Item	Dietary Trp supplementation				
	ZTS	LTS	HTS	SEM	*P* value
DAO	144.61^b^	138.55^b^	176.60^a^	1.81	0.006
D-lactate	14.50^b^	14.34^b^	15.68^a^	0.09	0.003

^a,b^Values with different letters within the same row are different (*P* < 0.05).

ZTS: zero Trp supplementation; LTS: low Trp supplementation; HTS: high Trp supplementation.

## References

[1] Miller B. G., James P. S., Smith M. W., Bourne F. J. (1986). Effect of weaning on the capacity of pig intestinal villi to digest and absorb nutrients. *The Journal of Agricultural Science*.

[2] Cera K. R., Mahan D. C., Cross R. F., Reinhart G. A., Whitmoyer R. E. (1988). Effect of age, weaning and post weaning diet on small intestinal growth and small intestinal morphology in young swine. *Journal of Animal Science*.

[3] Nabuurs M. J. A., Hoogendoorn A., van der Molen E. J., van Osta A. L. M. (1993). Villus height and crypt depth in weaned and unweaned pigs, reared under various circumstances in the Netherlands. *Research in Veterinary Science*.

[4] Pluske J. R., Williams I. H., Aherne F. X. (1996). Villous height and crypt depth in piglets in response to increases in the intake of cows' milk after weaning. *Animal Science*.

[5] Boudry G., Péron V., Le Huërou-Luron I., Lallès J. P., Sève B. (2004). Weaning induces both transient and long-lasting modifications of absorptive, secretory, and barrier properties of piglet intestine. *Journal of Nutrition*.

[6] Moeser A. J., Klok C. V., Ryan K. A. (2007). Stress signaling pathways activated by weaning mediate intestinal dysfunction in the pig. *American Journal of Physiology—Gastrointestinal and Liver Physiology*.

[7] Koopmans S. J., Ruis M., Dekker R., Van Diepen H., Korte M., Mroz Z. (2005). Surplus dietary tryptophan reduces plasma cortisol and noradrenaline concentrations and enhances recovery after social stress in pigs. *Physiology and Behavior*.

[8] Shen Y. B., Voilqué G., Kim J. D., Odle J., Kim S. W. (2012). Effects of increasing tryptophan intake on growth and physiological changes in nursery pigs. *Journal of Animal Science*.

[9] Adeola O., Ball R. O., House J. D., O'Brien P. J. (1993). Regional brain neurotransmitter concentrations in stress-susceptible pigs. *Journal of Animal Science*.

[10] Lepage O., Vílchez I. M., Pottinger T. G., Winberg S. (2003). Time-course of the effect of dietary L-tryptophan on plasma cortisol levels in rainbow trout oncorhynchus mykiss. *Journal of Experimental Biology*.

[11] Poletto R., Meisel R. L., Richert B. T., Cheng H.-W., Marchant-Forde J. N. (2010). Aggression in replacement grower and finisher gilts fed a short-term high-tryptophan diet and the effect of long-term human-animal interaction. *Applied Animal Behaviour Science*.

[12] Cirillo C., Vanden Berghe P., Tack J. (2011). Role of serotonin in gastrointestinal physiology and pathology. *Journal of Endocrine System Diseases*.

[13] Nylander O., Pihl L. (2006). Luminal hypotonicity increases duodenal mucosal permeability by a mechanism involving 5-hydroxytryptamine. *Acta Physiologica*.

[14] Yamada T., Inui A., Hayashi N., Fujimura M., Fujimiya M. (2003). Serotonin stimulates endotoxin translocation via 5-HT3 receptors in the rat ileum. *American Journal of Physiology-Gastrointestinal and Liver Physiology*.

[15] Wang H., Ji Y., Wu G. (2015). L-tryptophan activates mammalian target of rapamycin and enhances expression of tight junction proteins in intestinal porcine epithelial cells. *The Journal of Nutrition*.

[16] NRC (2012). *Nutrient Requirements of Swine*.

[17] Koopmans S. J., Guzik A. C., Van Der Meulen J. (2006). Effects of supplemental L-tryptophan on serotonin, cortisol, intestinal integrity, and behavior in weanling piglets. *Journal of Animal Science*.

[18] Martínez-Trejo G., Ortega-Cerrilla M. E., Rodarte-Covarrubias L. F. (2009). Aggressiveness and productive performance of piglets supplemented with tryptophan. *Journal of Animal and Veterinary Advances*.

[19] Li Y. Z., Kerr B. J., Kidd M. T., Gonyou H. W. (2006). Use of supplementary tryptophan to modify the behavior of pigs. *Journal of Animal Science*.

[20] Liu X., Feng J., Xu Z. R., Wang Y. Z., Liu J. X. (2008). Oral allergy syndrome and anaphylactic reactions in BALB/c mice caused by soybean glycinin and *β*-conglycinin. *Clinical and Experimental Allergy*.

[21] Chang X., Wang L. L., Lian S. J., Tang Q., Chen P., Wang H. (2012). Changes of endotoxemia rats in intestinal mucosal histology and plasma, intestinal tissue of two amine oxidase, plasma D-lactate. *Chin J Clin*.

[22] Ren W., Yin J., Wu M. (2014). Serum amino acids profile and the beneficial effects of L-arginine or L-glutamine supplementation in dextran sulfate sodium colitis. *PLoS ONE*.

[23] Shen L., Weber C. R., Raleigh D. R., Yu D., Turner J. R. (2011). Tight junction pore and leak pathways: a dynamic duo. *Annual Review of Physiology*.

[24] Li X., Akhtar S., Choudhry M. A. (2012). Alteration in intestine tight junction protein phosphorylation and apoptosis is associated with increase in IL-18 levels following alcohol intoxication and burn injury. *Biochimica et Biophysica Acta—Molecular Basis of Disease*.

[25] Jiang W.-D., Wen H.-L., Liu Y. (2015). The tight junction protein transcript abundance changes and oxidative damage by tryptophan deficiency or excess are related to the modulation of the signalling molecules, NF-*κ*B p65, TOR, caspase-(3,8,9) and Nrf2 mRNA levels, in the gill of young grass carp (*Ctenopharyngodon idellus*). *Fish and Shellfish Immunology*.

[26] Kim J. W., Lin H. C. (2007). Contribution of gut microbes to gastrointestinal motility disorders. *Practical Gastroenterology*.

[27] DeMeo M. T., Mutlu E. A., Keshavarzian A., Tobin M. C. (2002). The small intestine and nutrition: intestinal permeation and gastrointestinal disease. *Journal of Clinical Gastroenterology*.

[28] Kojima S.-I., Tohei A., Anzai N. (2012). A role for endogenous peptide YY in tachykinin NK2 receptor-triggered 5-HT release from guinea pig isolated colonic mucosa. *British Journal of Pharmacology*.

