# Erratum to “Effect of High Dietary Tryptophan on Intestinal Morphology and Tight Junction Protein of Weaned Pig”

**DOI:** 10.1155/2017/8309364

**Published:** 2017-10-16

**Authors:** Myrlene Carine B. Tossou, Hongnan Liu, Miaomiao Bai, Shuai Chen, Yinghua Cai, Veeramuthu Duraipandiyan, Hongbin Liu, Tolulope O. Adebowale, Naif Abdullah Al-Dhabi, Lina Long, Hussain Tarique, Abimbola O. Oso, Gang Liu, Yulong Yin

**Affiliations:** ^1^Key Laboratory of Agro-Ecological Processes in Subtropical Region, Institute of Subtropical Agriculture, Chinese Academy of Sciences, Hunan Provincial Engineering Research Center of Healthy Livestock, Scientific Observing and Experimental Station of Animal Nutrition and Feed Science in South-Central, Ministry of Agriculture, Hunan Co-Innovation Center of Animal Production Safety, Hunan 410125, China; ^2^University of Chinese Academy of Sciences, Beijing 100049, China; ^3^China Animal Disease Control Center, Beijing 102600, China; ^4^Department of Botany and Microbiology, Addiriyah Chair for Environmental Studies, College of Science, King Saud University, P.O. Box 2455, Riyadh 11451, Saudi Arabia; ^5^Department of Animal Nutrition, College of Animal Science and Livestock Production, Federal University of Agriculture, PMB 2240, Abeokuta, Nigeria

In the article titled “Effect of High Dietary Tryptophan on Intestinal Morphology and Tight Junction Protein of Weaned Pig” [1], there was an error in the in-text citations of Tables  4 and 5.

In Introduction, the text reading “However, little is known about the effect of dietary Trp on intestinal epithelial cells growth and health, as well as intestinal epithelial tight junction proteins in weaned piglets [7, 15] (Table  5)” should be corrected to “However, little is known about the effect of dietary Trp on intestinal epithelial cells growth and health, as well as intestinal epithelial tight junction proteins in weaned piglets [7, 15].”

Additionally, in “3.3. Effect of Tryptophan on Pig Intestinal Morphology,” the text reading “The morphology of the duodenum, jejunum, and ileum at 21 days of the experiment is shown in Table  4” should be corrected to “The morphology of the duodenum, jejunum, and ileum at 21 days of the experiment is shown in Table  5.”
